# Depressive symptoms among clients attending monk healers and primary care clinics in Thailand: a comparative follow-up study

**DOI:** 10.1017/S1463423621000475

**Published:** 2021-08-11

**Authors:** Supa Pengpid, Karl Peltzer

**Affiliations:** 1 ASEAN Institute for Health Development, Mahidol University, Salaya, Phutthamonthon, Nakhon Pathom, Thailand; 2 Department of Research Administration and Development, University of Limpopo, Polokwane, South Africa; 3 Department of Psychology, College of Medical and Health Science, Asia University, Taichung, Taiwan

**Keywords:** depressive symptoms, comparative study, primary care, monk healer, Thailand

## Abstract

**Background:**

The aim of this study was to conduct a comparative follow-up assessment of clients with depressive symptoms attending monk healers or primary care clinics in Thailand.

**Methods:**

Consecutively attending clients of three monk healing and three primary care centres who screened positive (a score of 9 or more) on the Primary Health Questionnaire (PHQ)-9 at the study site were followed up at 3 months after baseline assessment.

**Results:**

In 3 monk healer sites, 448 clients agreed to be screened with the PHQ-9 for depression, and 94 screened positive, and in 3 health centres 582 clients agreed to be screened, and 92 screened positive for depressive symptoms on the PHQ-9. In 2 monk healing sites, 79 clients (84%) were followed up at 3 months, and in 3 health centres, 79 clients (85.9%) were followed up at 3 months. At 3-month follow-up, mixed modelling found significant interaction effects (a time-by-condition interaction, i.e., between-group changes) on depression scores (*P* = <0.001). Depressive symptoms significantly decreased over time, but there was no significant difference in decline between the two groups.

**Conclusion:**

Clients attending monk healers decreased more in depressive scores compared to clients attending primary care centres, though there was no group effect with respect to number of depressed clients.

## Introduction

Traditional and faith healers form a major part of the mental health care workforce worldwide (Nortje *et al.*, 2016). In a study among patients with chronic disease, including mental disorders, out-patients in Thailand, 26.3% had been consulting traditional health practitioners in the past year (Peltzer *et al.*, 2016). ‘Traditional healers, including spiritual, herbal, and massage healers and traditional midwives, are distributed all over Thailand operating from their homes, religious institutions and health care facilities’ (Adthasit *et al.*, 2007). ‘Buddhist monks who are Thai traditional healers are called *maw pra* (monk healer) and they provide treatment, including Thai traditional medicine and indigenous practices, to the public at the Buddhist temple where they reside’ (Kaewla and Wiwanitkit, 2015; Chan-iam *et al.*, 2019). Several researchers (Jilek-Aall and Jilek, 1985; Phramaha Phanuvit Pannapajato, 2013) have described the utilization of monk healers for treating mental illness, and common mental and substance use disorders in Thailand.

There is a gap for the treatment of mental disorders in low- and middle-income countries (LMIC), including Thailand (Demyttenaere *et al.*, 2004). The World Health Organization (WHO) ‘mental health Global Action Programme Intervention Guide (mhGAP-IG)’ recognizes ‘non-specialists that include traditional and faith healers fall, are a potential resource to reduce the treatment gap in low-resource countries’ (WHO, 2010) and recommends to include traditional health practitioners in achieving the Global Mental Health Action plan (WHO, 2013). Nortje *et al.* (2016) conclude that evidence suggests that many persons in LMIC seek care from traditional health practitioners for mental health problems, and that traditional healer interventions ‘might help to relieve distress and improve mild symptoms in common mental disorders such as depression and anxiety’ (Nortje *et al.*, 2016). None of the reviewed studies by Nortje *et al.* (2016) had been conducted in Southeast Asia, including Thailand.

Studies conducted in Southeast Asian countries, including Thailand, for the treatment of mental and substance use disorders have been descriptive (Salan and Maretzki, 1983; Jilek-Aall and Jilek, 1985; Razali, 2000; Kurihara *et al.*, 2006; Khan *et al.*, 2010; Nguyen, 2014; Kaewla and Wiwanitkit, 2015). For example, in ‘Wat Thamkrabok, a Buddhist monastery in Thailand, has been conducting a drug addiction rehabilitation programme which claims a 70% success rate’ (Barrett, 1997). A few controlled studies integrated the traditional treatment into a controlled trial setting. For example, in a controlled study in a hospital setting in Thailand, an aerobic walking exercise incorporating the Buddhist meditations (*N* = 15) was found to be effective in reducing depression relative to a sedentary control group (*N* = 15) and traditional walking exercise training (*N* = 15) (Prakhinkit *et al.*, 2014). Rungreangkulkij *et al.* (2011) found in a controlled study a significant effect of Buddhist group therapy (*N* = 32) relative to a control group (standard physician treatment (*N* = 32) on type 2 diabetes patients with depressive symptoms in a hospital setting). However, both studies only incorporated Buddhist treatment elements into a medical treatment setting and suffered from small sample sizes, but no study has been conducted in Thailand on the religious health practitioner’s impact on the treatment of depressive disorders.

Previous comparative follow-up studies assessing the effectiveness of traditional health practitioner treatment for common mental disorders (depression, anxiety, somatization) used a ‘natural control’ setting, comparing the treatment of common mental disorders by traditional health practitioners and primary health care providers (Kleinman and Gale, 1982; Koss, 1987; Patel *et al.*, 1998). There is little comparative outcome research on the management of common mental disorders, such as depression, in Southeast Asia, including Thailand. In a comparative outcome study in Zimbabwe, the persistence of caseness of common mental disorder (assessed with the Shona Symptom Questionnaire) was at 2 and 12 months follow-up among traditional medical practitioner attenders 36% and 38%, respectively, and among primary health care attenders 48% and 43%, respectively (Patel *et al.*, 1998). In an uncontrolled outcome study among depressed traditional health practitioner attendees in Kenya, the Becks Depression Inventory score significantly decreased from 26.5 at baseline to 23.0 at 6 weeks follow-up and 17.2 at 12 weeks follow-up (Musyimi *et al.*, 2017).

Despite the existence of traditional health practitioners, research on the outcomes of the use of traditional and religious interventions to treat common mental disorders, such as depression, is non-existent in Southeast Asia, including in Thailand. Therefore, in filling this research gap, the aim of this study was to conduct a comparative follow-up assessment of clients with depressive symptoms attending monk healers or primary care clinics in Thailand. It was hypothesized that clients with depressive symptoms attending monk healers and primary care centres will have a significant reduction of depression symptoms at 3 months follow-up.

## Methods

### Sample and procedure

The study design is a longitudinal two-arm non-randomized intervention study of adult clients attending two different treatment settings, purposefully selected monk healers (*n* = 3) and primary care (*n* = 3) sites in two regions in Thailand. Clients attending any of the two treatment settings were consecutively screened by trained external interviewers with the Primary Health Questionnaire (PHQ)-9 and those who scored positive on the PHQ-9 (a score of 9 or more) were followed up at 3 months following baseline assessment from 2018 to 2019. Follow-up interviews took place at the study site and/or by phone. The questionnaire was translated and back-translated by bi-lingual researchers (apart from the PHQ-9 which had already been validated in Thailand by Lotrakul *et al.* (2008) into the study language Thai). The study questionnaire was pre-tested for validity on a sample of 30 clients, which did not form part of the final sample. The study received ethics approval from the ‘Office of The Committee for Research Ethics (Social Sciences), Mahidol University (No.: 2017/055.1403)’, and written informed consent was obtained from participants.

Sample size calculation was based on the absolute change in major depression symptoms as measured by the PHQ-9 from the pre-intervention to the 3-month follow-up assessment. The comparative study was powered to detect an effect size of 0.42 or larger on the PHQ-9 in a one-sided test (alpha = 0.05) at a power of 80%, and assuming a common standard deviation of the PHQ-9 scores of 8.4, a total of 150 (*n* = 75 in each group) (considering a loss to follow-up of up to 25%, *n* = 100 were needed in each group) (Cuijpers *et al.*, 2007).

### Intervention

In both treatment settings, monk healer and primary health care, routine care was provided to attending clients. In the monk healer setting, various treatment modalities can be applied, including prayer, herbal medicine, meditation, dietary interventions, making a merit, exorcism of evil spirits, practice the Dharma, removing bad *karma*, magic water treatment, admission to in-patient religious activities, individual and group counselling, and physical therapies (Jilek-Aall and Jilek, 1985; Phramaha Phanuvit Pannapajato, 2013; Kaewla and Wiwanitkit, 2015; Chan-iam *et al.*, 2019). In the primary care setting, ‘individuals who screen positive with the PHQ-2 screen and are assessed by the PHQ-9 scale to have mild or more serious depression are referred to a physician, in order for an accurate diagnosis to be made. Once a diagnosis is confirmed, in case of mild depression, psychological education and counselling is provided, and in case of moderate or severe depression the treatment consists of antidepressant medication, in addition to psychological interventions’ (Kongsuk *et al.*, 2017, p.35).

### Measures

The PHQ-9 was used to assess depressive symptoms (Kroenke *et al.*, 2001). The PHQ-9 showed ‘high sensitivity (0.84) and specificity (0.77) in a validation study in Thailand, using a cut-off score of nine or more as indicative for major depressive disorder’ (Lotrakul *et al.*, 2008). Cronbach’s alpha for the PHQ-9 was 0.88 in this study.

*Socioeconomic data* included subjective economic status (extent of debts), employment status, education, sex, age, marital status, and religion.

### Data analysis

Pearson chi-square tests were used for comparing categorical variables and nonparametric Mann–Whitney *U* tests for comparing continuous variables. The outcome (depression scores) was analysed using ‘intention-to treat principle for participants who had completed the required assessments both at the baseline and at a 3-month follow-up’. Of the in total 186 baseline study participants, 158 (85%) were followed up at 3 months and formed part of the analysis. The intervention effects on continuous 3-month outcomes (depression scores) and the prevalence of a positive screen for depressive symptoms on the PHQ-9 were calculated using gamma and logistic mixed-effect models adjusted for clustering (i.e., a random effect of site) and baseline covariates (gender, education, age, employment status, and economic status). Fixed effects comprised of condition, time, and a time-by-condition interaction (i.e., between-group changes) and baseline covariates. Mixed models were used because they most appropriately handle missing data (Schafer and Graham, 2002; O’Connel *et al.*, 2017). The data were analysed using ‘IBM-SPSS for Windows, version 25 (Chicago, IL, USA)’.

## Results

**Figure 1.**
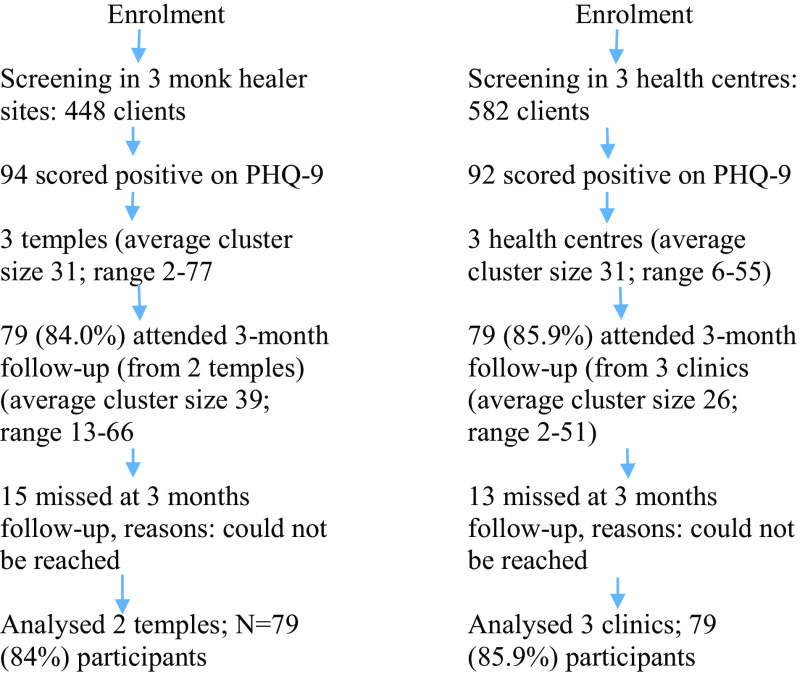
Study flow chart

Figure 1 summarizes client or patient identification, recruitment, and follow-up numbers. In 3 monk healer sites, 448 clients agreed to be screened with the PHQ-9 for depression, and 94 screened positive (a score of 9 or more), and in 3 health centres 582 clients agreed to be screened, and 92 screened positive for depressive symptoms on the PHQ-9. In 2 monk healer sites, 79 clients (84%) were followed up at 3 months, and in three health centres, 79 clients (85.9%) were followed up at 3 months (see Figure 1).

### Participant characteristics at baseline

Table 1 shows the sample characteristics in the intervention and control condition. In both treatment settings, majority were female and all were Buddhists by religion. Compared to primary care clients, monk healer clients had higher education and economic status, were younger in age, and had higher depression scores (see Table 1).

**Table 1.** Comparison of sociodemographic characteristics of intervention (*N* = 92) and control (*N* = 94)

	Intervention (Buddhist monk)	Control (health centre)	
	*N* = 92	*N* = 94	
Variable	Median (IQR)	Median (IQR)	*P*-value
Age in years	45 (18) (range: 28–81)	61 (17) (range 19–85)	<0.001
Depression scores	11 (5.25)	10 (3)	0.005
Sex, *N* (%)			
Female	81 (88.0)	70 (74.5)	0.065
Male	11 (12.0)	24 (25.5)	
Formal education			
Primary or less	37 (39.4)	81 (89.0)	<0.001
Secondary	26 (27.7)	6 (6.6)	
Post-secondary	31 (33.0)	4 (4.4)	
Marital status			
Never married	29 (30.9)	5 (5.5)	<0.001
Married	43 (45.7)	70 (76.9)	
Divorced/separated/widowed	22 (23.4)	16 (17.6)	
Religious affiliation			
Other	0 (0.0)	0 (0.0)	
Buddhist	92 (100)	94 (100)	
Employment status			
No	37 (39.4)	42 (45.7)	0.516
Yes	57 (60.6)	50 (54.3)	
In debt			
No	26 (27.7)	20 (21.7)	0.005
Little	40 (42.6)	24 (26.1)	
High	28 (29.8)	48 (52.2)	

### Depression outcomes

Table 2 shows the depressive symptoms study outcomes at baseline and 3-month post-assessment and findings of multilevel mixed modelling. At 3-month follow-up, mixed modelling found significant interaction effects on depression scores (*P* < 0.001). Finally, the prevalence of a positive screen for depressive symptoms on the PHQ-9 significantly decreased over time (from T1 to T2). Compared to health centre treatment, the monk healer intervention produced a higher reduction in the prevalence of a positive screen for depressive symptoms on the PHQ-9, but this was not statistically significant (see Table 2).

**Table 2.** Changes in depression scores and prevalence of screening positive on the PHQ-9

Variable	Clients of monk healer	Clients of health centre	Group effects	Time effects	Group × time effects
Depression scores	Median (IQR)	Median (IQR)	COE (SE)^1^	COE (SE)^1^	COE (SE)^1,^ ^2^
T1	11 (5.25)	10 (3)	−0.222 (0.058) *P* < 0.001	−0.828 (0.038) *P* < 0.001	−0.322 (0.069) *P* < 0.001)
T2	2 (4)	4 (4)			
	N (%)	N (%)	COE (SE)^3^	COE (SE)^3^	COE (SE)^2^,^3^
PHQ-9 (9 or more)					
T1	94 (100)	92 (100)	−0.263 (0.143) *P* = 0.066	−1.086 (0.272) *P* < 0.001	−0.295 (0.167) *P* = 0.078
T2	6 (7.6)	11 (13.6)			

COE = coefficient; SE = standard error.

1 Gamma mixed regression model.

2 Adjusted for cluster effects and baseline demographic variables.

3 Logistic mixed regression model.

## Discussion

Results suggest depressed clients decreased their depression scores in both groups, but more so among clients attending monk healer, than among primary health centre clients. These findings confirm results from previous studies in Africa (Patel *et al.*, 1998; Musyimi *et al.*, 2017) showing the potential efficacy of monk healers in the management of depressive symptoms. Monk healers may be better placed than time-pressured primary care practitioners to explore the psychosocial and spiritual basis as well as the wider family and social context of depressive symptoms and can enhance a ‘collective suggestion of a miraculous cure, cathartic abreaction and a symbolic process of the curing ritual’ (Jilek-Aall and Jilek, 1985). Future studies will need to be conducted to assess the components of the treatment, to explain further the potential treatment efficacy. Such studies may need to include methods, variables, and instruments that are relevant to the local socio-cultural context. Research results suggest increasing the dialogue between monk healers and health centre care providers to further improve the management of depressive symptoms in Thailand. Monk healers may have more to offer and could jointly participate in training programmes with primary health care workers and may want to undergo academic training and register as Thai traditional medicine practitioners (Peltzer and Pengpid, 2019).

### Study limitations

Depressive symptoms were only measured using a screening instrument for depression. Future studies should include a psychiatric assessment. It is possible that both clients attending monk healers and health centres were seeking care for symptoms different from depression and treatments provided were for problems other than depression. Further studies will be needed to establish the specific treatment methods used in the management of depressive symptoms as well as the time and costs involved in the treatment.

## Conclusion

This is the first comparative outcome study of clients with depressive symptoms attending monk healers and primary care centres in Thailand. The reduction in depression scores showed a better result for treatment by monk healers compared to treatment in primary health care centres, though number of depressed patients decreased similarly in both groups. Monk healers in Thailand may be included in the treatment of common mental disorders, such as depressive symptoms. This could be in the form of jointly participating in training programmes with primary health care workers, undergoing academic training and register as Thai traditional medicine practitioners. Further research, such as a randomized control trial and measurement of the direct effects of monk healing, is needed.
